# Social Value Orientation and Capitalism in Societies

**DOI:** 10.1371/journal.pone.0165067

**Published:** 2016-10-28

**Authors:** Shibly Shahrier, Koji Kotani, Makoto Kakinaka

**Affiliations:** 1 School of Economics and Management, Kochi University of Technology, Kochi-shi, Kochi, Japan; 2 Research Center for Future Design, Kochi University of Technology, Kochi-shi, Kochi, Japan; 3 College of Business, Rikkyo University, Toshima-ku, Tokyo, Japan; 4 Urban Institute, Kyushu University, Nishi-ku, Fukuoka, Japan; 5 Graduate School for International Development and Cooperation, Hiroshima University, Higashi-Hiroshima, Hiroshima, Japan; University of Westminster, UNITED KINGDOM

## Abstract

Cooperation and competition are core issues in various fields, since they are claimed to affect the evolution of human societies and ecological organizations. A long-standing debate has existed on how social behaviors and preferences are shaped with culture. Considering the economic environment as part of culture, this study examines whether the ongoing modernization of competitive societies, called “capitalism,” affects the evolution of people’s social preferences and behaviors. To test this argument, we implemented field experiments of social value orientation and surveys with 1002 respondents for three different areas of Bangladesh: (i) rural, (ii) transitional and (iii) capitalistic societies. The main result reveals that with the evolution from rural to capitalistic societies, people are likely to be less prosocial and more likely to be competitive. In a transitional society, there is a considerable proportion of “unidentified” people, neither proself nor prosocial, implying the potential existence of unstable states during a transformation period from rural to capitalistic societies. We also find that people become more proself with increasing age, education and number of children. These results suggest that important environmental, climate change or sustainability problems, which require cooperation rather than competition, will pose more danger as societies become capitalistic.

## Introduction

Competition and cooperation have been important issues in various fields, such as anthropology, biology, economics and sociology, because they are considered determinants of the evolution of human societies and ecological organizations [1, 2]. In evolutionary dynamics, competition is advantageous in the short run, but for long-run survival, cooperation can also be an effective strategy [1, 3]. In economics, rational self-interest models under competition can efficiently allocate private goods but cannot fully solve some public and intertemporal problems, such as natural resource allocation, public goods provision and resource sustainability for future generations [4, 5].

Social behaviors and preferences cannot be fully explained by genetic properties [3, 6–11]. Alternatively, culture-gene coevolutionary theory argues that human beings learn ideas and culture through a social learning mechanism, and this cultural transmission shapes human behaviors and preferences along with genetical properties [1, 3, 7, 11–17]. With culture-gene coevolutionary theory, the economic environment can be considered part of culture and is expected to affect people’s social preferences and behaviors. Given the economic growth of societies, together with concerns about environmental problems and future sustainability, this article addresses the relation between economic development and social preferences (or social behaviors) that are central to competition and cooperation in societies.

Several studies have documented how culture affects human behavior of competitiveness, fairness, equity and trust. Henrich et al. [7, 18] show that in indigenous societies, people exhibit higher prosociality and fairness when they are integrated into a market. Our research differs from that of Henrich et al. [7, 18] in that we study people and their social value orientations in three large-scale societies that are integrated into markets and have different degrees of capitalism, holding features such as language and religion constant. Leibbrandt et al. [2] show that fishermen in individualistic lake-based fisheries are more competitive than those in collective sea-based fisheries, suggesting that interactions with other people in the workplace affect human behaviors and preferences. Using a decomposed game of social value orientation (hereafter, SVO), Van Lange et al. [19] show that economics students are more competitive than psychology students and that “prosocial” individuals volunteer more in practice. Ockenfels and Weimann [20] and Brosig-Koch et al. [21] study people’s cooperative and solidarity behaviors in Eastern and Western Germany based on their different economic and social histories. They find that subjects from Eastern Germany act more selfishly than those from Western Germany in both public goods and solidarity games. By implementing a value-based study in 20 capitalistic countries, Schwartz [22] shows that people express stronger preferences for values such as self-assertiveness, mastery of natural and human resources, conformity, power and achievement in more market-driven and competitive societies.

None of the past studies focuses on the degree of capitalism in societies to analyze human behaviors and preferences for competition and cooperation despite the growth of capitalism that has taken place around the world. In this paper, we define “ongoing modernization of competitive societies” as capitalism and call highly modernized and competitive societies capitalistic. Most previous studies have been conducted in laboratories with student pools and in developed countries. Nevertheless, to generalize and understand real human behaviors, preferences and their implications, further studies are necessary in developing countries, as argued in Henrich et al. [23]. This study examines how the degree of capitalism in economic environments brings about an evolution in human behavior and social preferences by conducting field experiments in Bangladesh. There have been many arguments related to capitalism, such as Tonnies’ Gemeinschaft/Gesselschaft and Durkheim’s mechanical/organic solidarities, since the 19th century. We admit that what we call capitalism, “ongoing modernization of competitive societies,” overlaps with these ideas.

Competition for survival and success is a major idea in capitalism. Individuals in capitalistic societies survive, achieve or gain success by going through competitions where a person’s success usually involves having more wealth, status, prestige, among other traits. Specifically, we consider success to be gaining more wealth, status, or prestige than others. In capitalistic societies, people compete to gain such things. We also observe that human nature and social behaviors appear to be quite different based on our first-hand research experiences between rural and urban areas in various parts of Bangladesh. Therefore, we hypothesize that as societies become more capitalistic, the idea of “competition for survival and success” as a cultural trait or meme propagates from brain to brain due to success bias transmission through social learning mechanisms such that people become more competitive.

We have implemented field surveys and experiments in three areas of Bangladesh: (1) rural, (2) transitional and (3) capitalistic societies. Each possesses the same ethnicity, religion, language and so on, but differs with respect to the degree of capitalism. The capital city, Dhaka, is the most densely populated and is a highly capitalistic society, and there exists a sizable gap between Dhaka and rural areas with respect to the degree of capitalism [24]. In each area, we have collected socioeconomic information and identified subjects’ SVOs as of a (i) competitive, (ii) individualistic, (iii) prosocial or (iv) unidentified type, following Van Lange et al. [25, 26]. With this data, we characterize SVOs in relation to the degree of capitalism, as well as other socioeconomic factors, through statistical analyses.

## Study regions

**Fig 1:**
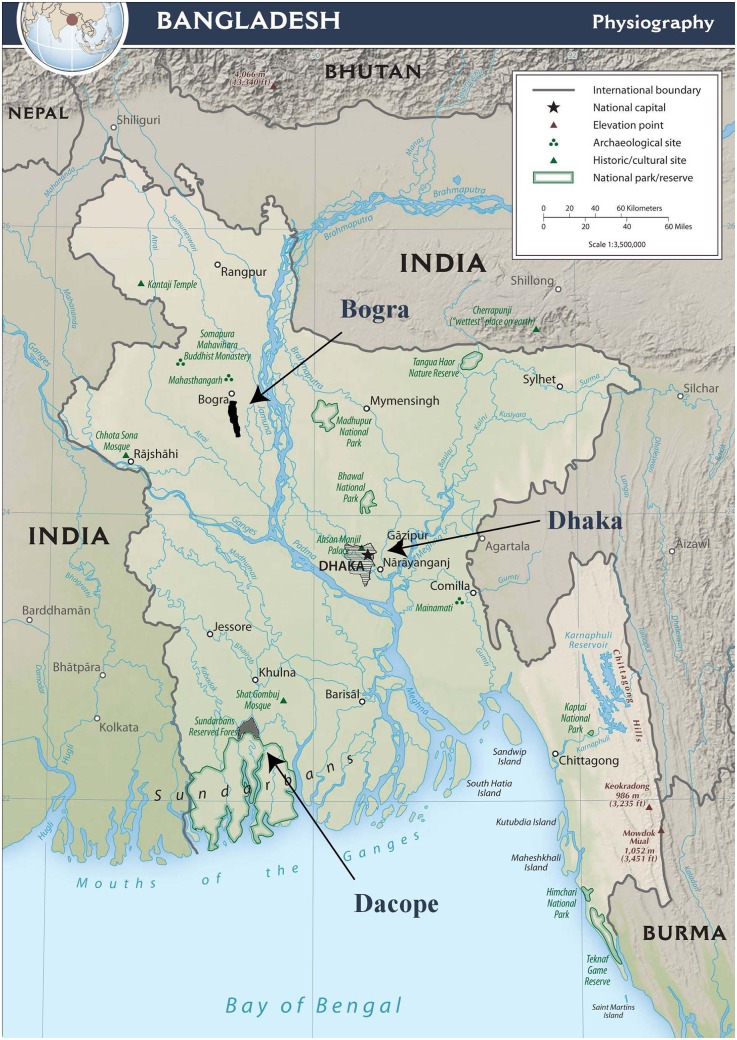
The three regions: Dhaka, Bogra and Dacope.

The field surveys and experiments have been implemented in three regions of Bangladesh: 1. Dhaka, the capital city (capitalistic), 2. several villages in the northern district Bogra (transitional) and 3. Dacope, a southern subdistrict (rural). Dhaka is the most densely populated and capitalistic city. Villages in Bogra have been gradually transforming from rural into capitalistic societies due to economic growth over the past few decades. Dacope is a rural area with the lowest level of capitalism, i.e., it is a highly ecosystem-based society. Bangladesh is ethnically and culturally homogeneous, and these three societies are all integrated into markets. They possess the same ethnic, language and religious variation. However, they differ from each other regarding the degree of capitalism. The locations are shown in Fig 1.

Dhaka city is located between 90°18’ and 90°57’ east longitude and 23°55’ and 24°81’ north latitude (Fig 1). The total land area, population and population density are 1371km^2^, 14.51 million and 10484 people km^−2^, respectively [24]. The population density in this region is almost nine times higher than that of the country average, and it is one of the most densely populated cities in the world [24]. Dhaka is the center of industrialization, business and service in Bangladesh. Business, service and some labor-intensive jobs are the major occupations in Dhaka. Few farming activities are available in the current Dhaka metropolitan area.

Several villages of the Shahjanpur subdistrict in the northern district of Bogra are located between 89°16’ and 89°29’ east longitude and 24°41’ and 24°50’ north latitude (Fig 1). The total land area and population density are 215.64km^2^ and 1307 people km^−2^, respectively. The population density is slightly higher than the country average of 1218 people km^−2^ [27]. Bogra is known as the gateway to the southern part of Bangladesh and as a modern and industrialized city. All the villages included in our survey and experiments have electricity and good communication with the nearest district city, Bogra. Modernization began with the efforts of several government agencies and NGOs to improve agriculture in that region. The Green Revolution, infrastructure development and suitable location for industrialization in Bogra led to its economic growth. Thus, this study region has been gradually transforming from a rural society into a capitalistic society. For simplicity, we refer to this study region as Bogra for the rest of the paper.

Dacope is located between 89°24’ and 89°35’ east longitude and 22°24’ and 22°40’ north latitude (Fig 1). The total land area of the Dacope subdistrict is 991.58 km^2^, and the population density is approximately 980 people km^−2^ [27]. The population density in Dacope is lower than the country average of 1218 people km^−2^. The infrastructure in this region is the least developed of the three study areas. The river network is the main channel of transportation. An earthen embankment was built to protect this region from storm surges, and it provides some limited road transportation. Except for some hatcheries and agriculture, there are few industries in this region.

Dacope is adjacent to the world’s largest mangrove forest, the Sundarbans. Unlike the other two study regions, business, farming and fishing are main occupations, which are contingent on the resources and rivers of the Sundarbans. Moreover, most of the households in this region are engaged in natural resource harvesting and agriculture for their self-consumption, apart from their main occupation for income earning. Due to the proximity of the world’s largest mangrove forest, as well as the absence of industries and service sectors, livelihoods in Dacope are dependent on nature and ecosystem services. Moreover, Dacope is located on the coastal belt of Bangladesh, which is one of the most lethal zones in the world due to storm hazards [28–30]. In summary, this society’s overall dependency on uncertain natural and ecosystem services is higher than that of Bogra and Dhaka.

## Methodology

### Social value orientation experiment

To measure people’s social preferences for competition and cooperation in three different regions, we employed a decomposed social value orientation (SVO) game developed by Van Lange et al. [25, 26]. The concept of SVO comes from a game-theoretical approach, which represents the effective matrix of outcomes for oneself and for another person [26]. In this game, numbers represent the outcomes for a pair, oneself and the other person, where the other person is unknown to the subject. Following Van Lange et al. [26], the game is called a triple-dominance decomposed game because each subject is asked to choose from among three options for one question. For example,

Option 1: You receive 500, and the other receives 100.
Option 2: You receive 500, and the other receives 500.
Option 3: You receive 560, and the other receives 300.

Option 1 represents a competitive orientation that maximizes the gap between oneself and the other (500 − 100 = 400) compared to any other option. Thus, subjects who choose option 1 can be considered competitive, as they seek to maximize their relative gain. Option 2 is a prosocial orientation that maximizes the joint outcome (500 + 500 = 1000). Finally, option 3 represents an individualistic orientation in that subjects who choose option 3 maximize their own outcome 560 and appear to be indifferent to the outcome of the other. The triple-dominant method of decomposed SVO games developed by Van Lange et al. [25, 26] consists of such nine questions, each of which consists of three options as introduced above. A major reason for using the triple-dominant method is its simplicity. Many subjects in these Bangladeshi areas are not educated, and we needed a simple game that everyone could understand. Subjects are asked to choose one of three options for each question and to answer nine total questions. The answers are first utilized to identify whether each subject’s orientation is competitive, individualistic or prosocial. Specifically, when at least 6 of 9 of the person’s choices are consistent with one of the orientations (competitive, individualistic and prosocial), he/she is categorized as that orientation. Otherwise, the subject is categorized as “unidentified.”

We implemented our experiments with monetary payments because we needed to attract people to the experimental sites and to encourage them to participate seriously, considering both transportation and opportunity costs. For each session, we collected 20 ∼ 40 subjects at a time in the experimental site. We gave the subjects instructions, and an experimenter (the first author) gave oral presentations to confirm the subjects’ understanding. Respondents were informed that the units in this game represented points and that the more points each respondent collected, the more real money he/she would earn from this game. To compute the respondent payoff from this game, we randomly matched respondents into pairs after eliciting their choices.

The experimental earnings from this SVO game were determined by summing the points earned from the 9 selections made for themselves and the 9 selections that their partner made for them, and an exchange rate was applied to determine the real monetary payment for each subject. We also explained the random matching method and payoff calculation using the exchange rate to determine the monetary payment received by the subjects. After eliciting the subjects’ answers in the SVO game, we conducted questionnaire surveys and collected each subject’s sociodemographic information. Each session took 40 ∼ 50 minutes, and the average payment was BDT 300 (≈ USD 3.30), with a show-up fee of BDT 150 (≈ USD 2.00).

### Random sampling in the field

We implemented different approaches to random sampling in the three study regions because they possess different sociodemographic and geographical characteristics. In each study region, we administered the field survey and experiments to 334 subjects. The surveys and experiments were administered mainly by the first author. The experiments were conducted between December 2014 and March 2015. All subjects are household heads or female subjects that earn income and make financial contributions to the household. In Dhaka, randomization was based on occupations to avoid overrepresentation of some specific groups of people. First, we computed the approximate proportion of each occupational category in the total population by referring to several governmental reports [27, 31]. Then, we randomly selected a number of organizations or companies for each category. We contacted the organizations, and based on their compliance, we randomly selected individuals from these organizations. However, for low-income occupations and occupations that require frequent movement within the city, such as rickshaw pullers and van drivers, we randomly selected slums and recruited the required number of people from among the residents of those slums. Our experiments were conducted in the classrooms at the Institute of Information Technology of Dhaka University.

In Bogra, we conducted household-level randomization. First, we determined the sample size based on the total number of households in each selected union. We conducted our experiments with 145, 99 and 90 subjects from the Aria Bazar, Amrool and Chupinagar unions, respectively, based on the number of households in each union. The number of households were collected from the respective local union offices. We randomly selected the household numbers and recruited income-earning members of households by sending them invitation letters. Finally, we were able to recruit enough subjects with our monetary incentives and invitation letters, and we conducted the experiments in several schools within the study region.

In Dacope, two unions were selected as the study sites, namely, Kamarkhola and Sutarkhali. The total number of households in Kamarkhola and Sutarkhali were 3559 and 7536, respectively [27, 31]. We randomly selected 108 (32% of the total subjects) and 226 (68% of the total subjects) subjects from Kamarkhola and Sutarkhali, respectively, based on the proportion of households in these two unions. Because a list of residents was not available from the local government office and people frequently move their shelters based on their daily activities, such as harvesting in the study region, we were unable to implement a typical randomization procedure for this region.

To implement random sampling in Dacope, we follow the procedure used in Himelein et al. [32, 33], called geographic cluster sampling. Prior to the experiments, we observed human traffic and the density of households within the study region using GIS technology. Moreover, we visited the study region twice before implementing the experiments. With the help of GIS technology and information obtained through field visits, we divided each of the unions into five subregions and divide each of the subregions into several seemingly equal strata with approximately the same number of households. Finally, we randomly selected an identical number of subjects from each stratum and invited them to participate in our experiments.

### Empirical method

We estimate a multinomial logit model to characterize the determinants of SVOs. Based on the SVO specifications, a subject falls into one of four orientations: (i) competitive, (ii) individualistic, (iii) prosocial and (iv) unidentified. The multinomial logit model is used to analyze the probability that a subject is assigned to an orientation, and it is specified as:

$$\begin{array}{c} {\text{Prob}}_{n}\left(i\right)=\text{Prob}\left(S_{in}\geq S_{In}\right),\;\forall I\neq i \end{array}$$ (1)

where *Prob*_*n*_(*i*) is the probability that subject *n* falls into orientation *i* of the four orientations *I* = {competitive, individualistic, prosocial, unidentified}. *S*_*in*_ is a function of independent variables that characterize the likelihood that a subject *n* falls into orientation *i*, which is specified as a linear form:

$$\begin{array}{c} S_{in}=\beta_{i}X_{n}+\epsilon_{in}, \end{array}$$ (2)

where **X**_*n*_ is a vector of independent variables, *β*_*i*_ is a vector of coefficients, and *ϵ*_*n*_ is a disturbance term that captures unobserved factors.

From Eqs (1) and (2), we can derive the following equation:

$$\begin{array}{ccc} {\text{Prob}}_{n}\left(i\right) & = & \text{Prob}(\beta_{i}X_{n}+\epsilon_{in}\geq \beta_{I}X_{n}+\epsilon_{In}),\;\forall I\neq i\\ & = & \text{Prob}(\beta_{i}X_{n}-\beta_{I}X_{n}\geq \epsilon_{In}-\epsilon_{in}). \end{array}$$ (3)

Eq (3) enables us to estimate coefficients of the independent variables by applying multinomial logit models. With this approach, Eq (3) reduces to the following closed form:

$$\begin{array}{c} {\text{Prob}}_{n}\left(i\right)=\frac{\exp\beta_{i}X_{n}}{\sum_{I}\exp\beta_{I}X_{n}}. \end{array}$$ (4)

The vector of coefficients *β*_*i*_ in Eq (4) can be estimated by standard maximum likelihood methods. The set of independent variables **X**_*n*_ includes household income, age, education, number of children under 12 years of age in the household, gender, family structure, occupation dummies and regional dummies.

Table 1 presents the definitions of the variables that are expected to be determinants of people’s SVOs. Age is coded as an ordered categorical variable from 0 to 5 following the work of Van Lange et al. [25] on the prosocial-growth or proself-growth hypothesis. The number of children under 12 and family structure are included since they are claimed to affect people’s social value orientations. Occupation dummies are included because the level of involvement in wage-labor occupations and dependence on ecosystem services is thought to affect social preferences [2, 18, 34, 35]. Specifically, we define the occupations as: (i) ecosystem service occupations (reference group), (ii) wage-labor occupations and (iii) business and service occupations. Ecosystem service occupations consists of farmers, fishermen and collectors of wood and honey from the forest. Wage-labor occupations comprise all construction workers, factory workers, van drivers and rickshaw pullers. Business and service occupations include all businessmen and job holders. Finally, regional dummies are included in the model to capture the effects of capitalism. The multinomial logit regression estimates the change in the probability that people are in a certain social value orientation when one independent variable is altered.

**Table 1:** Description of variables.

Variables	Description
SVO categories	Competitive, individualistic, prosocial and unidentified.
Household income	Household income per month in BDT 1000.
Age	Categorical variable of {0, 1, 2, 3, 4, 5} where ages between 20 and 29, 30 and 39, 40 and 49, 50 and 59, 60 and 69, and 70 and over are coded as 0, 1, 2, 3, 4 and 5, respectively.
Education	Years of schooling.
Children under 12	Number of children under 12 years of age in the household.
Gender	Dummy variable that takes 1 when the subject is male, otherwise 0.
Family structure	Single-family structures are coded as 1, otherwise (joint family) 0.
Occupation dummy	Ecosystem service occupation is the reference group. Two dummy variables are defined for wage-labor occupation and business & service, respectively.
Regional dummy	Dacope is the reference group. Two dummy variables are defined for Dhaka and Bogra.

One might assume reverse causality between SVOs and regional dummies. A large number of studies have analyzed the objectives and determinants of urban-rural migration. These studies find that economic difficulties such as poverty and unemployment are the main causes for rural-urban migration, and none of these studies suggests that competitive people tend to be attracted to capitalistic societies [36–39]. If reverse causality plays a role in our analysis, more competitive people should migrate to the most capitalistic region, Dhaka. Also, in the context of Bangladesh, it is reported that the majority of migrants to Dhaka are marginalized rural people and remain poor even after migration [24, 40, 41]. If these two facts are true at the same time, income would show a correlation with SVOs in our regression analysis. However, we have not found any correlation between value orientation and income, which we will show in the results section.

### Ethics statement

This study was approved by the research ethics committee of Kochi University of Technology. Subjects provided their written consent to participate in this study.

## Results

### Summary statistics

Table 2 provides the summary statistics for the independent variables. First, household income is the highest in Dhaka and the lowest in Dacope. This reflects the fact that Dhaka is a highly industrialized and capitalistic region, while Dacope is the least developed region where people’s livelihoods depend on ecosystem services. As mentioned in the previous section, Bogra can be considered in between. Thus, the household income data are consistent with our intuition. The gap between the rich and the poor seems to be the highest in Dhaka, since the standard deviation (hereafter, SD) of household income is the highest among the three regions.

**Table 2:** Summary statistics for the independent variables, 1002 observations (each region has 334 observations).

	Regions			Overall
	Dhaka	Bogra	Dacope	
Monthly household income in BDT 1000				
Average (Median)^1^	110 (35.00)	16 (12.00)	13 (10.00)	47 (15.00)
SD^2^	566	21	12	330
Min	3	3	2	2
Max	10000	350	100	10000
Age (ordered categories)^3^				
Average (Median)	0.66 (0.00)	1.58 (1.00)	1.53 (1.00)	1.26 (1.00)
SD	0.85	1.39	1.26	1.26
Min	0	0	0	0
Max	5	5	5	5
Education (years)				
Average (Median)	12.66 (16.00)	6.26 (5.00)	6.56 (5.00)	8.50 (10.00)
SD	5.30	4.96	4.57	5.76
Min	0.00	0.00	0.00	0.00
Max	20.00	17.00	17.00	20.00
Number of children (<12 year-old)				
Average (Median)	0.84 (1.00)	0.65 (1.00)	1.12 (1.00)	0.86 (1.00)
SD	1.08	0.78	0.90	0.95
Min	0.00	0.00	0.00	0.00
Max	6.00	6.00	4.00	6.00
Gender (female = 0)				
Average (Median)	0.82 (1.00)	0.95 (1.00)	0.93 (1.00)	0.90 (1.00)
SD	0.39	0.22	0.25	0.30
Min	0	0	0	0
Max	1	1	1	1
Family structure (joint family = 0)				
Average (Median)	0.62 (1.00)	0.75 (1.00)	0.46 (0.00)	0.61 (1.00)
SD	0.49	0.43	0.50	0.49
Min	0	0	0	0
Max	1	1	1	1
Occupation dummy (ecosystem service occupation = 0)				
Wage-labor occupation				
Average (Median)	0.22 (0.00)	0.27 (0.00)	0.24 (0.00)	0.43 (0.00)
SD	0.41	0.45	0.43	0.43
Min	0	0	0	0
Max	1	1	1	1
Business & service				
Average (Median)	0.78 (1.00)	0.39 (0.00)	0.53 (1.00)	0.57 (1.00)
SD	0.41	0.49	0.50	0.50
Min	0	0	0	0
Max	1	1	1	1

^1^ Median in parentheses.

^2^ SD stands for standard deviation.

^3^ The age variable is defined as an ordered categorical variable (Table 1).

The population of Dhaka is relatively younger than that of Bogra and Dacope. Nevertheless, the overall average age of 32.6 years suggests that most people in these three regions are of working age. In addition, people in Dhaka are highly educated, with 16 years of schooling, while most people in Bogra and Dacope have only 5 years of schooling. The average number of children under 12 years old in the three regions is highest in Dacope, and the number of joint family dwellings is significantly higher in Dacope than in Dhaka and Bogra. Furthermore, the summary statistics of occupations show that the sample in Dhaka consists only of wage-labor occupations and business and service occupations. On the other hand, in Bogra and Dacope, ecosystem service, wage-labor and business & service occupations are well represented, so Bogra and Dacope rely more on ecosystem services than Dhaka.

The ecosystem service occupations consist of only farming in Bogra, while it comprises farming and natural resource harvesting, such as wood and honey collecting, in Dacope. Unlike Bogra, almost 100% of households in Dacope are engaged in subsistence farming and natural resource harvesting for their self-consumption in addition to their main occupation. Since these activities are not income-generating, we do not consider these their occupations. This dependency on ecosystem services in Dacope has been captured by the regional dummy. All of these summary statistics are consistent with our expectations, including the ordering of the three regions in the degree of capitalism. With the lowest population density, the lowest average household income, the highest number of joint family households, the highest average number of children per household and the highest dependency on ecosystem services, Dacope is the least capitalistic society followed by Bogra and then Dhaka.

Table 3 presents the summary statistics for subjects’ SVOs across the three regions. The number of competitive people is the highest in Dhaka (32.34%), the next-highest in Bogra (23.65%) and the lowest in Dacope (17.66%). In addition, individualists are the largest group in Dacope (32.63%) followed by Dhaka (30.84%) and Bogra (22.46%). Moreover, 31.74%, 19.16% and 15.27% of subjects are classified as having an “unidentified” value orientation in Bogra, Dhaka and Dacope, respectively. Finally, the number of prosocial subjects is highest in Dacope (34.43%) and smallest in Dhaka (17.66%). Overall, the results provide clear evidence that competitive and prosocial people are dominant in Dhaka and Dacope, respectively, whereas the proportion of unidentified people is outstanding in Bogra. This tendency seems to suggest that in a transitional society, such as Bogra, people’s SVOs could be unstable, while people in Dhaka and Dacope reflect a tendency toward competitive and prosocial orientations, respectively.

**Table 3:** Percentage of each social value orientation by study region (*N* = 1002, 334 observations per region).

	Competitive	Individualistic	Unidentified	Prosocial
Dhaka	32.34	30.84	19.16	17.66
Bogra	23.65	22.46	31.74	22.16
Dacope	17.66	32.63	15.27	34.43
Overall	24.55	28.64	22.06	24.75

### Social value orientation in relation to the degree of capitalism

First, on the basis of Table 3, we conduct pair-wise chi-squared tests of the categorical variables for the three regions to see whether the distribution of SVOs is independent of the regions. Specifically, the null hypothesis is that the distributions of SVOs are identical for any two regions. We confirmed that the results of all possible pairs (Dhaka & Dacope, Dhaka & Bogra and Dacope & Bogra) reject the null hypothesis at a 1% level of significance and *χ*^2^(3) > 20, which suggests that the distribution of SVOs is dependent upon the regions. Therefore, the societies wherein people reside might influence their SVOs, controlling for other factors.

To establish our result, we next estimate two different multinomial logit regressions and one logistic regression. Table 4 presents the marginal effects for models 1 and 2 from multinomial logit regressions. In model 1, we include all independent variables, along with the regional dummy variables, except for the occupation dummies. To check the robustness of our results, model 2 includes the occupation dummies, with the consideration of the possibility that the degree of individual involvement in wage-labor occupations and dependence on ecosystem services might affect their social value orientations. In addition, Table 5 presents the marginal effects from the logistic regression (model 3). In this model, we drop all observations of individualistic and unidentified social value orientations to clarify the difference between prosocial and competitive orientations with respect to the regional dummies, controlling for all other independent variables. The results show the marginal probabilities that subjects are in the competitive, individualistic and unidentified orientations relative to being in the reference group of prosocial actors when an independent variable changes.

**Table 4:** Models 1 and 2: marginal effects of a multinomial logit regression with prosocial as the reference group (*N* = 1002).

	Model 1			Model 2		
	Competitive	Individualistic	Unidentified	Competitive	Individualistic	Unidentified
Monthly household income (in BDT 1000)	0.000	0.000	−0.000	0.000	0.000	−0.000
	(0.000)	(0.000)	(0.000)	(0.000)	(0.000)	(0.000)
Education (years of schooling)	0.011***	−0.001	−0.010***	0.012***	−0.002	−0.011***
	(0.003)	(0.003)	(0.003)	(0.003)	(0.003)	(0.003)
# of children (< 12 years old)	−0.019	0.027*	0.025*	−0.021	0.026*	0.025*
	(0.017)	(0.016)	(0.015)	(0.017)	(0.016)	(0.015)
Male (base group = female)	0.029	0.069	0.071*	0.031	0.069	0.070*
	(0.043)	(0.047)	(0.040)	(0.045)	(0.047)	(0.040)
Age (categorical variables)	0.022*	−0.004	−0.007	0.025**	0.004	−0.009
	(0.012)	(0.014)	(0.011)	(0.012)	0.014)	0.011)
Single family (base group = joint family)	0.012	−0.038	0.014	0.010	−0.039	0.016
	(0.029)	(0.032)	(0.029)	(0.030)	(0.032)	(0.029)
Regional dummy (base group = Dacope)						
Dhaka	0.096**	−0.022	0.102***	0.085**	−0.021	0.110***
	(0.044)	(0.040)	(0.042)	(0.044)	(0.041)	(0.042)
Bogra	0.053	−0.101***	0.163***	0.053	−0.098***	0.164***
	(0.038)	(0.035)	(0.038)	(0.039)	(0.035)	(0.038)
Occupation dummy (base group = ecosystem service occupation)						
Wage-labor occupation				0.056	−0.008	−0.038
				(0.051)	(−0.008)	(0.038)
Business & service				0.005	0.024	−0.002
				(0.044)	(0.045)	(0.039)

The Wald *χ*^2^ statistic is 102.67 and 104.09 for multinomial logit model 1 and model 2, respectively, and significant at the 1 percent level.

***significant at the 1 percent level

**significant at the 5 percent level and

*significant at the 10 percent level.

**Table 5:** Model 3: marginal effects of the logit regression with prosocial as the reference group (*N* = 494).

	Competitive
Monthly household income (in BDT 1000)	0.00
	(0.00)
Education (years of schooling)	0.012*
	(0.01)
# of children (< 12 years old)	0.007
	(0.031)
Male (base group = female)	0.167
	(0.076)
Age (categorical variables)	0.027
	(0.022)
Single family (base group = joint family)	0.001
	(0.052)
Regional dummy (base group = Dacope)	
Dhaka	0.279***
	(0.072)
Bogra	0.182***
	(0.059)
Occupation dummy (base group = ecosystem service occupation)	
Wage-labor occupation	0.022
	(0.077)
Business & service	0.007
	(0.070)

The Wald *χ*^2^ statistic is 39.83 for the logit regression, significant at the 1 percent level.

***significant at the 1 percent level and

*significant at the 10 percent level.

We first provide a quick overview of our results and then explain the detailed results based on model 1 in Table 4. Household income and family structure have no explanatory power for SVOs. The coefficient on education is statistically significant for the competitive and unidentified orientations. The number of children under 12 in a household affects the relative likelihood of being in the individualistic and unidentified groups. Relative to being in the prosocial group, gender positively affects the probability of being in the unidentified group, and age positively affects the probability of being in the competitive. Finally, the regional dummies for Dhaka and Bogra are significant predictors of being in the competitive, individualistic and unidentified groups, compared to being in the prosocial group, taking Dacope as the reference group.

Concerning household income, the insignificant result in Table 4 is consistent with the previous studies [1, 2, 7, 18], which report that income is not a determinant of behavior associated with competitiveness, fairness, equity or trust. Regarding family structure, some studies argue that higher interdependence at the family level leads to prosocial orientation [19, 25]. Thus, we initially expected that the joint family structure may induce people to be in the prosocial group, since it is naturally associated with more interactions with relatives and family members. However, our analysis demonstrates that family structure does not affect the probability of being in a specific value orientation. This result is consistent with the argument of Leibbrandt et al. [2] that interdependence at the social level rather than at the family level is a significant determinant of competitiveness.

The effects of education in model 1 of Table 4 suggest that an additional year of education increases the likelihood of being in the competitive group by 1.1% and decreases the likelihood of being in the unidentified group by 1.0%, relative to being in the prosocial group. It should be noted that one-standard-deviation increase in education (approximately 6 years, i.e., high school plus university) affects the likelihood of being in the competitive and unidentified groups by 6.37% and −5.76%, respectively. Our results suggest that the education system in Bangladesh influences individuals’ likelihood of being proself with the number of years of schooling. The current Bangladeshi education system requires young people to engage in cutthroat competition for admission to good high schools and universities. We conjecture that such severe competition in Bangladeshi education is one reason for the increased likelihood of being in the competitive group and the decreased likelihood of being in the unidentified group with longer periods of schooling. Education contains the same cultural trait or meme as capitalism, that is, “competition for survival and success,” which could propagate from brain to brain through a social learning mechanism [3, 7, 14]. As people spend more years in a competitive education system, they become more competitive [3, 14, 15].

The results in model 1 of Table 4 also show that having one more child under 12 in a household increases the probability of being in the individualistic and unidentified groups by 2.7% and 2.5%, respectively, relative to being in the prosocial group. This finding is in contrast with the argument in Van Lange et al. [25] that people become more prosocial with more interaction and experience with children. However, this difference may reflect the special context of a developing country like Bangladesh, which may be very distinct from that of a developed country. A possible explanation may be that raising a young child in a developing country often comes with more difficulties and hardships than in a developed country. An adult needs to work hard for his/her children and even sacrifices himself/herself to ensure their daily survival. In such a situation, the probability of being in the individualistic or unidentified group is expected to increase with each additional child in a household.

With respect to the significant result related to the gender dummy, the probability of being in the unidentified group is higher for males than for females by 7.1% relative to being in the prosocial group. Van Lange et al. [25] find that females are more prosocial than males. However, our finding suggests that females’ social preferences are more deterministic than those of males. Furthermore, regarding the age effect, the empirical analysis shows a 2.2% rise in the probability of being in the competitive group relative to being in the prosocial group when age increases by one 10-year category. This result conflicts with the prosocial-growth hypothesis, which is claimed in Van Lange et al. [25]. Instead, it seems to support the proself-growth hypothesis. The magnitude of the age effect might be considered significant, because individual preferences related to competitiveness and cooperation are known to change very slowly after the early stage of their life [7, 21, 42]. Developing countries such as Bangladesh have neither social security systems nor other public support for elderly people. Therefore, elderly people are required to compete for a stable future as they age, which may cause their social preferences to be more competitive.

Now, we closely look at how SVOs differ across regions. Recall that in the rural society (Dacope), the “prosocial” group is dominant. On the other hand, in the capitalistic society (Dhaka), “competitive” people are dominant. In the in-between society (Bogra), the portion of people in the unidentified group becomes larger. As expected, the results related to the regional dummies in model 1 confirm that individuals in Dhaka are more likely to be in the competitive group by 9.6% than those in Dacope relative to being in the prosocial. Likewise, individuals in Dhaka and Bogra are more likely to be in the unidentified group by 10.2% and 16.3%, respectively, than those in Dacope relative to being in the prosocial group. Individuals in Bogra are less likely to be in the individualistic group by 10.1% than those in Dacope as compared to being in the prosocial group. We can verify the robustness of our results, since the same qualitative results are observed in model 2 in Table 4.

As a further robustness check, model 3 in Table 5 reports the result of the logit regression, focusing on the difference between the prosocial and competitive value orientations across regions. Compared to those in Dacope, individuals in Dhaka and Bogra are 27.9% and 18.2% more likely to be in the competitive group, respectively, than in the prosocial group. This result illustrates a clear change from prosocial to competitive value orientations with respect to the degree of capitalism in societies. That is, as societies become more capitalistic, people tend to become less prosocial and more competitive. As part of this robustness check, we estimate a logistic regression between competitive and prosocial orientations with the regional dummy as the only independent variable. We also conducted a contextual/hierarchical analysis using a multilevel multinomial logit regression. Both of these analyses confirm the same qualitative results that have been presented in this paper. For simplicity, we have not included the results in the paper, but they are available upon request. All three models (models 1, 2 and 3) exhibit the same qualitative results, which suggest that the economic environment, i.e., the degree of capitalism in the society, is a crucial determinant of SVOs.

As mentioned earlier, competition for survival and success is one of the main ideas in capitalism. Individuals in capitalistic societies survive, achieve, or succeed by competing. Hence, we argue that with aging capitalism in societies, the idea of “competition for survival and success” as a cultural trait or meme transfers from brain to brain due to success bias transmission through a social learning mechanism such that people become more competitive. We can provide one simple example of how competition for survival and success as a cultural trait engenders human behaviors: competition for buses in Dhaka. Many people usually wait at bus stops in Dhaka. When a bus approaches the stop, it is usually filled with more passengers than its capacity. Only a few more people can take the bus on each occasion. Therefore, people waiting at the stop compete to get on the bus by pushing and pulling each other. In this case, survival and success means that each person goes home as early as possible. There are two ways to survive and achieve success: (1) forming a first-come and first-served queue and (2) competing by pushing and pulling at a bus stop. Unfortunately, in Dhaka, pushing and pulling to get on a bus is a common behavior due to the propagation of competition for survival and success.

Surprisingly, the proportion of people in the “unidentified” group in Dacope (the least capitalistic society) is the lowest among the three regions, while it is the highest in the transitional society of Bogra. This result implies the potential existence of unstable states in people’s social preferences. Previous studies of SVOs, such as Van Lange et al. [19, 25, 26], do not pay attention to the existence of such “unidentified” subjects. A gradual change in economic environment is plausibly one reason for the large number of unidentified value orientations in Bogra. In Bogra, cooperation for survival and success was dominant in the past, as is common in rural, agrarian societies. However, as the society gradually becomes capitalistic, the new cultural trait of competition for survival and success appears to conflict with the old pattern. Thus, the conflict seems to bring about instability in people’s preferences as “unidentified.” This temporary instability may also imply the gradual transformation of an individual’s social preference from prosocial to competitive, and it is likely that with the aging of capitalism in this society, people will become more competitive.

It is worth describing some of our observations of the real-life economic practices in Dacope that make people more prosocial. Our survey data confirm that unlike Dhaka and Bogra, almost 100% of the households in Dacope are engaged in farming and natural resource harvesting for their self-consumption apart from their main occupation for income earning. These activities are unique characteristics of daily life in Dacope and are absent in the other two societies. Hence, in Dacope, economic activities require cooperation rather than competition to ensure mutual long-term survival under natural uncertainty and hardship. In Dacope, people enter the adjacent forest, the Sundarbans, to collect wood or honey, and they need to cooperate for ensuring their safety from wild animals, such as tigers. Moreover, it is common to share the profits or goods equally, regardless of how much wood or honey they collect individually. The same type of sharing practices can be seen among the fishermen who harvest together in adjacent rivers. Due to the existence of such cooperative practices and needs, in the long run, cooperation for survival and success is still dominant. As a consequence, people in this region are more prosocial than those in Dhaka and Bogra, which is consistent with the finding in Leibbrandt et al. [2].

Finally, we check whether individuals have enough interactions with others in everyday life and whether such experiences of social interactions reflect the individual social preferences identified in our experiments [13, 25, 43]. Specifically, we hypothesize that people’s interactions with neighbors in each society have some association with their value orientations, because interactions with neighbors seem to change with the transformation of societies. Interactions with friends have been difficult to quantify on the same basis between rural and urban areas because these interactions are heterogeneous based on their different environments and factors such as the availability of the internet. Moreover, interactions with friends are somewhat dependent upon each individual’s personality and reflect the fact that friends are chosen endogenously but neighbors are exogenously given in Bangladeshi societies. Therefore, we use “interaction with neighbors” as the main instrument.

We collected individual information about the frequency of interactions with neighbors. To avoid an endogeneity problem between value orientation and social interaction, we have not included the frequency of interactions with neighbors as an independent variable in the regression. Table 6 presents the summary statistics for the frequency of interacting with neighbors per month in each region. Interestingly, the distribution of the frequency of interactions with neighbors and the SVOs exhibit the same qualitative tendency with respect to the regional dummies. People in Dacope interact with their neighbors most frequently among our three study regions, as captured by both the average and the median. On the other hand, people in Dhaka have the lowest frequency, based on the average and the median. People in Bogra fall in between. The standard deviations reveal the same tendency, except that Bogra’s standard deviation is bit higher than Dacope’s. Overall, it appears that economic development changes interactions with neighbors and social preferences. That is, as societies become more capitalistic, people are less likely to interact with their neighbors. This result is also consistent with those of our regression analyses.

**Table 6:** Frequency of interactions with neighbors per month (*N* = 1002, 334 observations per region).

Frequency of interactions per month	Regions			Overall
	Dhaka	Bogra	Dacope	
Average	12.7	28.6	30.3	23.9
Median	4	18	30	15
Standard deviation	15.4	31.9	27.8	27.2
Min	0	0	0	0
Max	120	200	150	200

To the best of our knowledge, this paper is the first to focus on the degree of capitalism in field experiments and to demonstrate that as societies become more capitalistic, people tend to be less prosocial and more competitive. The literature has already documented the reliability of culture-gene coevolution [1–3, 7, 11, 12, 16–18]. Our results can be considered additional evidence of culture-gene coevolution when we interpret capitalistic economic development as part of culture. Our results suggest that important environmental, climate change or sustainability problems, wherein cooperation rather than competition is necessary, shall be more pressing as societies become more capitalistic.

## Conclusion

The literature shows that culture can bring about evolutions in human behaviors and preferences. Considering that competition for survival and success is one of the main ideas in capitalism, it is likely that, with economic development and maturation of capitalism in societies, people learn the idea of competition for survival and success and tend to be more competitive. Hence, we have analyzed individual social preferences in relation to the degree of capitalism in societies. Most of the previous studies that address the issue of coevolution between humans and culture have been conducted in laboratories or in developed countries. The field experiments in Bangladesh enable us to study social preferences in relation to the degree of capitalism, since Bangladesh has a wide gap between rural and capitalistic societies.

Our analysis demonstrates that with evolution from rural to capitalistic societies, people are likely to be less prosocial and more likely to be competitive. In a transitional society, there is a considerable proportion of people in the “unidentified” group, which is neither proself nor prosocial, implying the potential existence of unstable states during the transformation period from rural to capitalistic societies. We argue that with maturing capitalism in societies, the cultural trait, i.e., an idea of “competition for survival and success,” propagates from one individual to another individual due to success bias transmission through a social learning mechanism, so people become less prosocial and more competitive. We have also found that people become more competitive with increasing age, education and number of children. These results suggest that important problems, such as environment, climate change or sustainability issues, where cooperation rather than competition is necessary, shall be more pressing as societies become more capitalistic.

We note some limitations of our study. We have tried to collect rich data on interactions among people that might be correlated with social value orientations (SVOs). Unfortunately, the economic environment seems to affect the way people interact each other, and some of our initial attempts proved impossible, such as quantifying interactions with friends and the quality of human relationships. In addition, our analyses and results could have been explored in various ways. For instance, we do not analyze SVOs in relation to people’s involvement and interactions in distinct frameworks such as traditional solidarity vs. anomic city life. We do not compare our results of the SVO games with other value measures, such as assertiveness, harmony, achievement, conformity and power orientation, used in Schwartz [22]. Since our analysis is based on the SVO games in the field, framing in the experiments may have affected the results as well. Thus, to confirm the robustness of the analysis, it is important to compare our experimental findings with those of different approaches. Future research should be able to account for such issues.

These caveats notwithstanding, it is our belief that this study provides a first step to addressing how the economic environment, specifically, the degree of capitalism in societies, brings about a change (or evolution) in people’s social preferences. The more societies develop under capitalism, the more the idea of competition for survival and success seems to propagate as cultural trait. More generally, this study provides an illustration of culture-gene coevolution in relation to capitalism with the idea of competition for survival and success as a cultural trait or meme as suggested by previous studies [3, 11, 12, 14, 16].

## Supporting Information

S1 File Excel “SVOs” data file. It contains all the necessary data to replicate the statistical and regression results presented in this paper.
(XLSX)
